# Cost-effectiveness of Finger Replantation Compared With Revision Amputation

**DOI:** 10.1001/jamanetworkopen.2019.16509

**Published:** 2019-12-02

**Authors:** Alfred P. Yoon, Tanvi Mahajani, David W. Hutton, Kevin C. Chung

**Affiliations:** 1 Section of Plastic Surgery, Department of Surgery, University of Michigan Medical School, Ann Arbor; 2 School of Public Health, University of Michigan, Ann Arbor; 3 Health Management and Policy, University of Michigan School of Public Health, Ann Arbor

## Abstract

**Importance:**

Traumatic digit amputation is the most common type of amputation injury, but the cost-effectiveness of its treatments is unknown.

**Objective:**

To assess the cost-effectiveness of finger replantation compared with revision amputation.

**Design, Setting, and Participants:**

This economic evaluation was conducted using data from the Finger Replantation and Amputation Challenges in Assessing Impairment, Satisfaction, and Effectiveness (FRANCHISE), a retrospective, multicenter cohort study at 19 centers in the United States and Asia that enrolled participants from August 1, 2016, to April 12, 2018. Model variables were based on the FRANCHISE database, Centers for Medicare & Medicaid Services, and published literature. A total of 257 participants with unilateral traumatic finger amputations treated with revision amputation or replantation distal to the metacarpophalangeal joint and at least 1 year of follow-up after treatment were included in the analysis.

**Exposures:**

Revision amputation or replantation of traumatic finger amputations.

**Main Outcomes and Measures:**

Main outcome measures were quality-adjusted life-years (QALYs), total costs (in US dollars), and incremental cost-effectiveness ratios (ICERs). A willingness-to-pay threshold of $100 000 per QALY was used to assess cost-effectiveness.

**Results:**

Of the 257 study participants (mean [SD] age, 46.7 [15.9] years; 221 [86.0%] male), 178 underwent finger replantation and 79 underwent revision amputation. In a base case of a 46.7-year-old patient, replantation was associated with QALY gains of 0.30 (95% credible interval [CrI], −0.72 to 1.38) for single-finger (not thumb), 0.39 (95% CrI, −1.00 to 1.90) for thumb, 1.69 (95% CrI, −0.13 to 3.76) for multifinger excluding thumb, and 1.27 (95% CrI, −2.21 to 5.04) for multifinger including thumb injury patterns. Corresponding ICERs for replantation compared with revision amputation were $99 157 per QALY for single-finger (not thumb), $66 278 per QALY for thumb, $18 388 per QALY for multifinger excluding thumb, and $21 528 per QALY for multifinger including thumb injury patterns. Sensitivity analysis revealed that age at time of injury, life expectancy, postinjury utility, wages, and time off work for recovery had the strongest associations with cost-effectiveness. Probabilistic sensitivity analysis revealed the following chances of replantation being cost-effective: 47% in single-finger (not thumb), 52% in thumb, 78% in multifinger excluding thumb, and 64% in multifinger including thumb injury patterns.

**Conclusions and Relevance:**

With proper patient selection, replantation of all finger amputation patterns, whether single-finger or multifinger injuries, may be cost-effective compared with revision amputation. Multifinger replantations had a higher probability of being cost-effective than single-finger replantations. Cost-effectiveness may depend on injury pattern and patient factors and thus appears to be important for consideration when patients and surgeons are deciding whether to replant or amputate.

## Key Points

**Question:**

Is replantation after traumatic finger amputation cost-effective compared with revision amputation?

**Findings:**

In this economic evaluation of data on 257 adults from the Finger Replantation and Amputation Challenges in Assessing Impairment, Satisfaction, and Effectiveness study, the incremental cost-effectiveness ratio for replantation compared with revision amputation was $99 157 in single-finger (not thumb), $66 278 in thumb, $18 388 in multifinger excluding thumb, and $21 528 in multifinger including thumb injury patterns per quality-adjusted life years.

**Meaning:**

With proper patient selection, replantation of all finger amputation patterns, whether single-finger or multifinger injuries, may be cost-effective compared with revision amputation.

## Teaser

This economic evaluation compares the cost-effectiveness of finger replantation vs revision amputation after traumatic finger amputation.

## Introduction

Traumatic finger amputation represents more than 90% of all amputations in the United States and has a yearly incidence of 45 000.^1,2^ This injury disproportionately affects younger working class individuals who incur considerable economic burden and disability.^3,4^ Traumatic finger amputations are treated with replantation or revision amputation. Replantation applies microsurgical techniques to reattach digital nerves, arteries, and veins, as well as bone fixation and tendon repairs to restore finger length, sensation, and function. Revision amputation shortens the finger while achieving wound closure; this mode of treatment is less costly, is less complex, and requires less postoperative therapy than replantation.

Previous studies^4,5,6,7,8,9^ have evaluated the clinical outcomes associated with replantation and revision amputation. However, there is a dearth of evidence on their cost-effectiveness. To our knowledge, the only economic analysis comparing the 2 treatments is reported in the study by Sears et al,^10^ in which a decision tree model based on a time trade-off survey of healthy individuals was used. That study^10^ concluded that the number of fingers amputated is substantially associated with the cost-effectiveness of replantation. However, a limitation of that study was that the time trade-off survey was based on a healthy population, which may have biased and inaccurately assessed disease burden. Given the increasing scrutiny of health care expenditures and pressure to attain maximal clinical efficacy with minimal expense, cost-effectiveness is essential to consider in clinical practice.

We aimed to perform a cost-utility analysis of finger replantation and revision amputation with use of data from an international collaborative group. To our knowledge, this is the only cost-effectiveness study to compare finger replantation and revision amputation using data from a patient cohort who sustained traumatic finger amputations. We evaluated the most pertinent factors associated with cost-effectiveness of finger replantation to assist in the decision-making process of traumatic finger amputation management.

## Methods

### Study Population and Data Source

This economic evaluation used data from the Finger Replantation and Amputation Challenges in Assessing Impairment, Satisfaction, and Effectiveness (FRANCHISE) study, which recruited adult participants from 19 US and Asian international centers between August 1, 2016, and April 12, 2018. The research protocol was approved by the participating sites’ local institutional review boards. Separate institutional review board exemption for the present study was obtained from the University of Michigan before analysis commenced. All human participants gave written informed consent in their native languages before the start of the FRANCHISE study. The consent obtained included consent to the use of the FRANCHISE data in future planned studies, and all data were deidentified. All unilateral traumatic finger amputations treated with revision amputation or replantation distal to the metacarpophalangeal joint were eligible. Patients who could not provide consent, underwent nontraumatic amputations, sustained bilateral amputations, or had amputations proximal to the metacarpophalangeal joint were excluded. Patients were asked to evaluate their outcomes with 4 patient-reported outcomes instruments at least 1 year postoperatively: Michigan Hand Outcomes Questionnaire; Disabilities of the Arm, Shoulder and Hand; Patient-Reported Outcomes Measurement Information System Upper Extremity Module; and Medical Outcomes Study 36-Item Short-Form Health Survey (SF-36), version 2. Data were collected in a database established by the Plastic Surgery Foundation using research electronic data capture.^11^ This study followed the Consolidated Health Economic Evaluation Reporting Standards (CHEERS) reporting guideline.

### Model Design

We performed a model-based economic analysis from a societal perspective. A decision tree was constructed based on finger injury pattern, procedure type, stages of recovery, and potential complications with a lifetime horizon. The SF-36 patient-reported outcomes for each procedure and injury type from the FRANCHISE database were used to determine the long-term health outcomes associated with each decision tree end point.

The base case scenario was a 46.7-year-old adult (based on the mean age in FRANCHISE) who sustained a single finger (not thumb) traumatic finger amputation and underwent an uncomplicated replantation. Other scenarios considered were thumb-only revision amputation vs replantation, multifinger excluding thumb revision amputation vs replantation, and multifinger including thumb revision amputation vs replantation. Patients first incur the costs of the initial procedure, recovery, and rehabilitation, which vary by procedure type (Table 1). Life expectancy was modeled as a dynamic function of age at time of injury based on the Centers for Disease Control and Prevention National Center for Health Statistics mortality data.^36^ The postoperative time off work for recovery and mean complication rates were based on literature review. Only complications that require operative intervention were included because they differed the most between the 2 treatments. Five potential complications were identified that can affect cost utility: neuroma, vascular reexploration, bone revision procedure, tendon revision procedure, and other minor revision procedures. Probabilities of these complications are given in Table 1.^4,5,7,8,9,13,14,15,16,17,18,19,20,21,22,23,24,25,26,27,28,29,30,31,32,33^

**Table 1.** List of Model Variables for Base Case Scenario^a^

Variable	Base Case Value	Low^b^	High^b^	Distribution	Source
Age at time of injury, y	46.7	18^c^	79^c^	Uniform	FRANCHISE database
Life expectancy, y	82	64	94	NA	Centers for Disease Control and Prevention^12^
**Complication Rates**					
Revision amputation, %					
Neuroma excision	6.4	0	30.0	β	Hattori et al,^8^ 2006; Jones et al,^9^ 1982; Hattori et al,^13^ 2003; Bouma et al,^14^ 2018; Harris et al,^15^ 2018; and van den Berg et al,^16^ 2012
Tendon procedure	7.5	0	15.0	β	Jones et al,^9^ 1982
Minor revision procedure	7.8	0	10.0	β	Jones et al,^9^ 1982; Harris et al,^15^ 2018; and Harvey and Harvey,^17^ 1974
Replantation, %					
Neuroma excision	3.0	0	10.0	β	Hahn and Jung,^7^ 2006; Hattori et al,^8^ 2006; and Scott et al,^18^ 1981
Vascular reexploration	13.9	0	40.0	β	Sebastin and Chung,^4^ 2011; Chang et al,^5^ 2015; Cavadas et al,^19^ 2018; Agarwal et al,^20^ 2010; Berlin et al,^21^ 2014; Scott et al,^18^ 1981; Sears and Chung,^22^ 2011; Datiashvili et al,^23^ 2007; Fufa et al,^24^ 2013; Hattori et al,^13^ 2003; Kamarul et al,^25^ 2018; Kim et al,^26^ 2018; Morrison et al,^27^ 1977; Nazerani et al,^28^ 2011; Ngaage et al,^29^ 2018; Rosberg,^30^ 2014; Shale et al,^31^ 2013; and Waikakul et al,^32^ 2000
Bone procedure	8.0	0	10.0	β	Hahn and Jung,^7^ 2006; Jones et al,^9^ 1982; Scott et al,^18^ 1981; and Yu et al,^33^ 2003
Tendon procedure	15.9	0	30.0	β	Jones et al,^9^ 1982; Scott et al,^18^ 1981; Fufa et al,^24^ 2013; and Yu et al,^33^ 2003
Minor revision procedure	17.9	0	25.0	β	Jones et al,^9^ 1982; Scott et al,^18^ 1981; Fufa et al,^24^ 2013; and Yu et al,^33^ 2003
**Costs**					
Revision amputation					
Direct costs, physician fees, $					
Single finger (not thumb)	703	597.55	808.45	Normal	Medicare (*CPT* codes 26951 and 26952)
Thumb only	703	597.55	808.45	Normal	Medicare (*CPT* codes 26951 and 26952)
Multifinger excluding thumb	2108	1791.80	2424.20	Normal	Medicare (*CPT* codes 26951 and 26952)
Multifinger including thumb	2108	1791.80	2424.20	Normal	Medicare (*CPT* codes 26951 and 26952)
Anesthesia fees	260	221.00	299.00	Normal	Medicare
Hospital fees	2811	2389.35	3232.65	Normal	Medicare and HCUP^34^
Medication cost	615	522.75	707.25	Normal	Sears et al,^10^ 2014
Physical therapy cost					
Single finger (not thumb)	2482	2109.70	2854.30	Normal	Medicare^d^
Thumb only	5904	5018.40	6789.60	Normal	Medicare^d^
Multifinger excluding thumb	6888	5854.80	7921.20	Normal	Medicare^d^
Multifinger including thumb	9840	8364.00	11 316.00	Normal	Medicare^d^
Family member wages lost, $					
Single digit	2162	1837.70	2486.30	Normal	US Census Bureau^35^
Thumb only	2162	1837.70	2486.30	Normal	US Census Bureau^35^
Multidigit excluding thumb	2853	2425.05	3280.95	Normal	US Census Bureau^35^
Multidigit including thumb	2853	2425.05	3280.95	Normal	US Census Bureau^35^
Replantation					
Direct costs, physician fees, $					
Single finger (not thumb)	2284	1941.40	2626.60	Normal	Medicare (CPT 20816)
Thumb only	2284	1941.40	2626.60	Normal	Medicare (CPT 20827)
Multifinger excluding thumb	6853	5825.05	7880.95	Normal	Medicare (CPT 20816)
Multifinger including thumb	6853	5825.05	7880.95	Normal	Medicare (CPT 20816)
Anesthesia fees					
Single finger (not thumb)	450	382.50	517.50	Normal	Medicare
Thumb only	450	382.50	517.50	Normal	Medicare
Multifinger excluding thumb	639	543.15	734.85	Normal	Medicare
Multifinger including thumb	639	543.15	734.85	Normal	Medicare
Hospital fees	14 737	12 526.45	16 947.55	Normal	Medicare and HCUP (DRG 906)^34^
Medication cost	615	522.75	707.25	Normal	Sears and Chung,^22^ 2011
Physical therapy cost					
Single finger (not thumb)	7199	6119.15	8278.85	Normal	Medicare^d^
Thumb only	7199	6119.15	8278.85	Normal	Medicare^d^
Multifinger excluding thumb	9840	8364.00	11 316.00	Normal	Medicare^d^
Multifinger including thumb	9840	8364.00	11 316.00	Normal	Medicare^d^
Family member wages lost, $^c^					
Single finger (not thumb)	2853	2425.05	3280.95	Normal	US Census Bureau^35^
Thumb only	2853	2425.05	3280.95	Normal	US Census Bureau^35^
Multifinger excluding thumb	2853	2425.05	3280.95	Normal	US Census Bureau^35^
Multifinger including thumb	2853	2425.05	3280.95	Normal	US Census Bureau^35^
Complication costs, $					
Neuroma excision					
Physician fee	693.00	589.05	796.95	Normal	Medicare (*CPT* code 64766)
Anesthesia fee	166.00	141.10	190.90	Normal	Medicare
Hospital fee	4918.00	4180.30	5655.70	Normal	Medicare
Physical therapy or rehabilitation	421.00	357.85	484.15	Normal	Medicare^e^
Family member wages lost	778.00	661.30	894.70	Normal	Medicare
Vascular reexploration					
Physician fee	1376.00	1169.60	1582.40	Normal	Medicare (*CPT* code 35231)
Anesthesia fee	450.00	382.50	517.50	Normal	Medicare
Hospital fee	2649.00	2251.65	3046.35	Normal	Medicare and HCUP^34^
Physical therapy or rehabilitation	0	0	0	Normal	Medicare^e^
Family member wages lost	519.00	441.15	596.85	Normal	US Census Bureau^35^
Bone procedure					
Physician fee	1079.00	917.15	1240.85	Normal	Medicare (*CPT* code 26546)
Anesthesia fee	260.00	221.00	299.00	Normal	Medicare
Hospital fee	5958.00	5064.30	6851.70	Normal	Medicare and HCUP^34^
Physical therapy or rehabilitation	0	0	0	Normal	Medicare^e^
Family member wages lost	2853.00	2425.05	3280.95	Normal	US Census Bureau^35^
Tendon procedure					
Physician fee	792.00	673.20	910.80	Normal	Medicare (CPT 26390)
Anesthesia fee	260.00	221.00	299.00	Normal	Medicare
Hospital fee	5958.00	5064.30	6851.70	Normal	Medicare and HCUP^34^
Physical therapy or rehabilitation	2335.00	1984.75	2685.25	Normal	Medicare^e^
Family member wages lost	4323.00	3674.55	4971.45	Normal	US Census Bureau^35^
Minor revision procedure					
Physician fee	98.00	83.30	112.70	Normal	Medicare (CPT 11042)
Anesthesia fee	166.00	141.10	190.90	Normal	Medicare
Hospital fee	330.00	280.50	379.50	Normal	Medicare and HCUP^34^
Physical therapy or rehabilitation	421.00	357.85	484.15	Normal	Medicare^e^
Family member wages lost	778.00	661.30	894.70	Normal	US Census Bureau^35^
**Wage Calculations**					
Annual wage before treatment, mean, $	40 248.00	10 000.00	250 000.00	Normal	FRANCHISE database and US Census Bureau^35^
Time off for recovery and rehabilitation					
Revision, d	50	28	10 000	Log normal	Goldner et al,^6^ 1990; Harris et al,^15^ 2018; Harvey and Harvey,^17^ 1974; and van den Berg et al,^16^ 2012
Replantation, d	120	90	10 000	Log normal	Goldner et al,^6^ 1990; Hattori et al,^8^ 2006; and Scott et al,^18^ 1981
Reduction in wages after recovery					
Revision, %	75.0	0	100	Normal	Assumption
Replantation, %	75.0	0	100	Normal	Assumption
Retirement age, y	68	57.8	78.2	Normal	Assumption
Discount rate, %	3.0	0.0	5.0	NA	Assumption
**Utilities**					
Revision amputation					
Single finger (not thumb)	0.845	0.807	0.883	Normal	FRANCHISE database (and 95% CI)
Thumb only	0.821	0.774	0.867	Normal	FRANCHISE database (and 95% CI)
Multifinger excluding thumb	0.733	0.662	0.805	Normal	FRANCHISE database (and 95% CI)
Multifinger including thumb	0.741	0.601	0.881	Normal	FRANCHISE database (and 95% CI)
Replantation					
Single finger (not thumb)	0.859	0.831	0.886	Normal	FRANCHISE database (and 95% CI)
Thumb only	0.838	0.795	0.882	Normal	FRANCHISE database (and 95% CI)
Multifinger excluding thumb	0.808	0.769	0.846	Normal	FRANCHISE database (and 95% CI)
Multifinger including thumb	0.798	0.716	0.880	Normal	FRANCHISE database (and 95% CI)

Abbreviations: *CPT*, *Current Procedural Terminology*; DRG, diagnosis related group; FRANCHISE, Finger Replantation and Amputation Challenges in Assessing Impairment, Satisfaction, and Effectiveness; HCUP, Healthcare Cost and Utilization Project; NA, not applicable.

^a^ The distributions for the probabilistic sensitivity analysis are parameterized as follows. All distributions are set to have approximately 95% of the distribution between the low and high values. The β distributions are based on 100 data points informing the estimate and using a noninformative prior. All normal distributions are truncated so they are greater than 0 (and <1 for the reduction in wages and utilities). All distributions are assumed to be independent.

^b^ For sensitivity analysis.

^c^ Varied based on age at time of injury.

^d^ On the basis of *CPT* codes 97165 or 97167, 97110, 97535, 97760, 97530, 97763, 97140, 97010, and 97018.

^e^ On the basis of 1 primary caregiver.

### Health States

Health is defined in terms of quality-adjusted life-years (QALYs), which are a function of level of health (health utility) over time (years). Health utility of each injury scenario and procedure type was derived from SF-36 surveys in FRANCHISE based on the methods developed by Brazier and Roberts^12^ (Table 2). Because the SF-36 data were collected at least 1 year from the participant's latest hand surgery, we assumed that the patient's health state remained stable for the remaining years of life. In addition, we assumed that the potential long-term negative effects in function or patient satisfaction from complications are reflected in the SF-36–derived utilities. The remaining years of life were based on the age at the time of injury and US mean life expectancy published by the Centers for Disease Control and Prevention^36^ (Table 1). Age at time of injury varied from 18 to 79 years, reflecting the participants' ages in FRANCHISE.

**Table 2.** Quality-Adjusted Life-years and ICERs by Finger Amputation Pattern

Injury Pattern^a^	SF-36 Converted Health Utility (95% CI)	*P* Value^b^	QALYs (95% CrI)^a^	Incremental QALYs (95% CrI)^a^	Total Cost Without Complications (95% CrI), $^a^	Incremental Costs (95% CrI), $^a^	ICER, $/QALYs^c^
Single-finger (no thumb) revision amputation	0.85 (0.80 to 0.88)	.57	19.25 (3.51 to 24.81)	0.3 (−0.72 to 1.38)	172 854 (9873 to 1 210 811)	29 425 (−739 256 to 809 853)	96 668
Single-finger (no thumb) replantation	0.86 (0.83 to 0.89)		19.56 (3.56 to 25.16)		202 279 (30 156 to 1 238 216)		
Thumb-only revision amputation	0.82 (0.77 to 0.87)	.68	18.7 (3.41 to 24.21)	0.4 (−1 to 1.9)	176 276 (13 101 to 1 213 553)	26 003 (−739 256 to 809 853)	64 614
Thumb-only replantatiom	0.84 (0.79 to 0.88)		19.1 (3.46 to 24.69)		202 279 (30 240 to 1 238 817)		
Multifinger excluding thumb revision amputation	0.73 (0.66 to 0.80)	.84	16.71 (3.07 to 22.07)	1.69 (−0.13 to 3.76)	179 356 (15 980 to 1 217 102)	30 322 (−735 706 to 813 301)	17 926
Multifinger excluding thumb replantation	0.81 (0.77 to 0.85)		18.4 (3.32 to 23.75)		209 678 (37 337 to 1 247 660)		
Multifinger including thumb revision amputation	0.74 (0.60 to 0.88)	.60	16.88 (3.03 to 23.32)	1.3 (−2.21 to 5.04)	182 308 (18 701 to 1 221 045)	27 370 (−738 236 to 811 792)	20 988
Multifinger including thumb replantation	0.80 (0.72 to 0.88)		18.18 (3.31 to 24.12)		209 678 (37 345 to 1 246 076)		

Abbreviations: CrI, credible interval; ICER, incremental cost-effectiveness ratio; QALY, quality-adjusted life-year; SF-36, Medical Outcomes Study 36-Item Short-Form Health Survey.

^a^ On the basis of model and probabilistic sensitivity analysis.

^b^ Amputation vs replantation.

^c^ Figure 3 gives uncertainty in cost-effectiveness.

### Costs

The model included the following direct costs: physician fee, anesthesia fee, hospital fee, medication cost, physical therapy cost, and complication costs. Indirect costs included the patients’ and family members’ wages lost during recovery. All costs were determined using Medicare reimbursement rates, the Healthcare Cost and Utilization Project from the Agency for Healthcare Research and Quality,^34^ and literature review. All cost data were adjusted for inflation to 2018 dollars using the gross domestic product deflator.

Physician and facility fees were derived from the 2018 National Physician Fee schedule using *Current Procedural Terminology* (*CPT*) codes. For single-finger and thumb-only revision amputation procedures, a mean of *CPT* codes 26951 and 26952 were used. For 2-, 3-, and 4-finger revision amputation procedures, we doubled, tripled, and quadrupled the costs by multiplying the 1-finger cost by the total number of fingers involved to approximate the variation in reimbursement. For replantation, we used *CPT* code 20816 for 1-, 2-, 3-, and 4-finger replantation and 20827 for thumb replantation. Anesthesia costs were drawn from Medicare reimbursements and were accounted for the initial procedures and complications. Hand therapy costs were provided by hand therapists at Michigan Medicine based on *CPT* codes (Table 1).

We included patients’ wages lost during and after recovery as indirect costs. We estimated time off work of 50 days for revision amputation and 120 days for replantation.^6,8,16,17,18,37^ These times were presumed to be the earliest these patients could return to work after these procedures. We hypothesized that 80% of traumatic finger amputations would be in workers in construction and extraction, farming, and fishery, estimating an annual wage of $31 508. The other 20% would be in workers in remaining occupations, such as food and service, office and administrative service, and protective service occupations. A weighted mean of these fields yielded a total annual mean wage of $40 248.^38^ Patients undergoing replantation and revision amputation often return to modified work; therefore, we assumed that everyone returned to modified work, with decreased retirement income at 65 years of age. In the base case, we speculated that patients’ wages after recovery from replantation and revision amputation were 75% of initial wages. Cost and health outcomes were discounted by 3%.

### Statistical Analysis

The primary outcome measure of this study was the incremental cost-effectiveness ratio (ICER) between replantation and revision amputation. This ratio was calculated by dividing the difference in total cost between the 2 procedures by the difference in QALY gained. We initially performed the base case calculation using mean values of the clinical variables in FRANCHISE. We then performed sensitivity analyses by varying key clinical, utility, and cost factors to determine which factors would have the strongest association with the ICER. Health utility scores were varied by the 95% CI of our data set. Remaining clinical variables (age at time of injury, postinjury wages,^35^ time off work, life expectancy,^36^ and complication rates) were varied based on sources listed in Table 1. Cost of care was varied by 15% from Medicare rates to account for differences among insurers. We reported the threshold values of key clinical variables that would be associated with increased ICER of replantation above a willingness-to-pay threshold of $100 000 per QALY.^39,40^ Two-way sensitivity analysis was performed by varying the difference in the most important key clinical variables between the 2 procedure types, determined by 1-way sensitivity analyses of all model variables. A probabilistic sensitivity analysis was conducted to simulate uncertainty in all parameters simultaneously. To accomplish this, we used a Monte Carlo simulation with 10 000 iterations of randomly varied inputs in the model. We used those simulation results to calculate 95% credible intervals (CrIs). An a priori significance level was set at *P* = .05. Statistical package R, version 3.6.0 (R Foundation for Statistical Computing) and Excel (Microsoft Inc) were used for modeling and analysis.

## Results

A total of 257 participants (mean [SD] age, 46.7 [15.9] years; 221 [86.0%] male) participated in the study. Of these patients, 178 underwent finger replantation and 79 underwent revision amputation. Baseline clinical factors between the 2 cohorts, including injured hand laterality, insurance status, sex, and educational level, were not significantly different. More nonwhite patients than white patients underwent replantation (159 [89.3%] vs 19 [10.7%], *P* = .02), and patients undergoing replantation were younger than those undergoing revision amputation (mean [SD] age, 45.3 [15.0] vs 49.8 [17.3] years; *P* = .04). The overall utility score difference between white and nonwhite patients was not significantly different (0.79 [95% CI, 0.75-0.83] vs 0.83 [95% CI, 0.81-0.85], *P* = .11). The revision amputation cohort had significantly more single-finger injury patterns than the replantation cohort (47 [59.5%] vs 67 [37.6%], *P* = .002). Health utility scores were higher for those with single-finger injuries (0.85 [95% CI, 0.82-0.87]) vs multifinger injuries (0.79 [95% CI, 0.75-0.82]) (*P* = .001). The mean health utility score was 0.01 to 0.08 points higher for patients undergoing replantation compared with patients undergoing revision amputation depending on injury pattern (Table 2). However, there was substantial variation in utilities among individuals, and the utility differences between the 2 cohorts were not significantly different.

Additional subgroup analyses of demographic variables between patients undergoing replantation and those undergoing revision amputation in each injury pattern group are given in eTable 1 in the Supplement. The revision amputation and replantation cohorts in the multifinger excluding thumb group and multifinger including thumb injury group had similar baseline characteristics, including age, sex, educational level, insurance status, and race. More nonwhite patients than white patients underwent replantation in the single finger (not thumb) group (67 [100%] vs 0 [0%], *P* < .001), and patients undergoing replantation tended to be younger than their counterparts (mean [SD] age, 45.6 [15.8] vs 52.1 [16.4] years; *P* = .04). In the 36 patients in the thumb-only injury cohort, the patients undergoing replantation had lower education levels (16 [44.4%] vs 0 [0%] with less than a high school education, *P* = .03) and were more likely to be nonwhite individuals (28 [77.8%] vs 8 [22.2%], *P* = .03) compared with the patients undergoing revision amputation (eTable 1 in the Supplement).

Lifetime QALYs were higher, but costs were also higher for replantation than revision amputation (Table 2 and the eFigure in the Supplement). The ICER of replantation compared with revision amputation was $99 157 in single-finger (not thumb), $66 278 in thumb-only, $18 388 in multifinger excluding thumb, and $21 528 in multifinger including thumb injuries.

**Figure 1.**
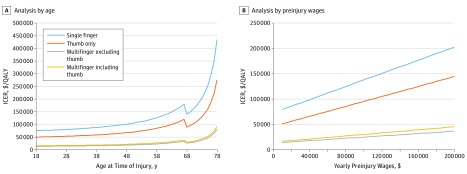
One-Way Sensitivity Analyses of Incremental Cost-effectiveness Ratio (ICER) Between Replantation and Revision Amputation QALY indicates quality-adjusted life-year.

The ICER was under the willingness-to-pay threshold ($100 000 per QALY) for patients younger than 48 years for single-finger (not thumb) injury, younger than 63 years for thumb-only injury, and 78 years for multifinger injuries (Figure 1A). Because the preinjury wages increased, replantation became less cost-effective because of costs incurred during rehabilitation. For single-finger (not thumb) amputations, replantation was cost-effective for preinjury wages under $42 000. For thumb-only amputations, the salary cutoff was approximately $109 000. For multifinger injuries, replantation remained cost-effective for preinjury wages above $200 000 (Figure 1B). We conducted 1-way sensitivity analyses on all model parameters to assess the uncertainties in cost-effectiveness estimates (eTable 2 in the Supplement).

**Figure 2.**
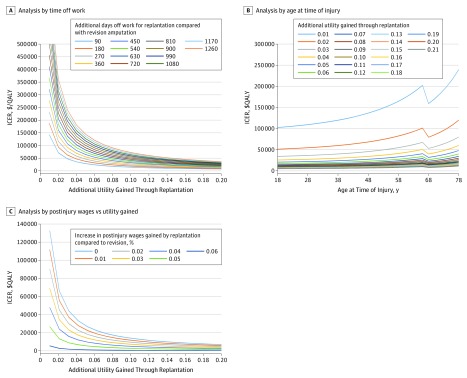
Two-Way Sensitivity Analyses Comparing the Difference In Model Variables Between Replantation and Revision Amputation For the 2-way sensitivity analysis between percentage difference in postinjury wages and additional utility gained with replantation, replantation became dominant (higher quality-adjusted life-years [QALYs] and lower societal costs) regardless of the amount of utility gain if postinjury replantation wages were more than 6% higher than those of revision amputation. ICER indicates incremental cost-effectiveness ratio.

The relative differences of model variables between replantation and revision amputation were associated with altered cost-effectiveness; thus, 2-way sensitivity analyses of the differences in time off work and utility were conducted. As time off work after replantation increased, replantation became less cost-effective. If the utility difference of replantation vs revision amputation was higher, replantation was more cost-effective. For younger patients between 20 and 40 years of age, even if patients took 6 to 7 months more time off work after replantation compared with revision amputation, when there is a utility difference of at least 0.02, replantation would still be cost-effective (Figure 2A). Two-way sensitivity analysis between age at time of injury and difference in health utility between replantation and revision amputation revealed that replantation would not be cost-effective if the utility difference was below 0.01 in any injury pattern or age group. However, if the utility difference was higher or equal to 0.02, replantation would be cost-effective for all injury patterns in patients younger than 65 years. Two-way sensitivity analysis of differences in postsurgery wages and differences in postsurgery health utility between the 2 treatments found that if there was a 2% or greater increase in postsurgery salary with replantation compared with revision amputation, replantation would be cost-effective even with a utility increase of 0.01 for all injury patterns (Figure 2B). Replantation is cost-saving to society if postsurgery salary with replantation is more than 6% higher than with revision amputation (Figure 2C).

**Figure 3.**
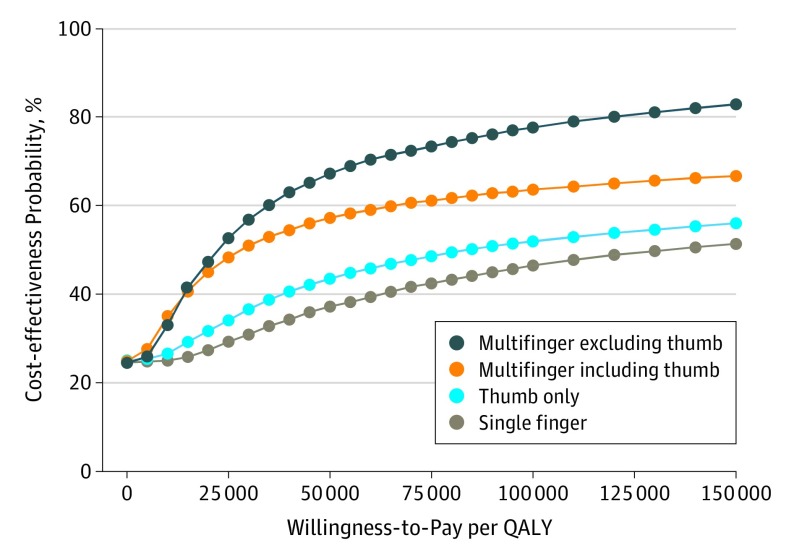
Probabilistic Sensitivity Analysis With 10 000 Iterations by Injury Pattern

Probabilistic sensitivity analysis with 10 000 iterations revealed that the chance of being cost-effective at a willingness-to-pay threshold of $100 000 per QALY would be 47% for single-finger (not thumb) replantation, 52% for thumb-only replantation, 78% for multifinger excluding thumb replantation, and 64% for multifinger including thumb replantation (Figure 3).

## Discussion

This study suggests that replantation in single-finger and multifinger injury patterns including or excluding thumb involvement can be cost-effective in certain settings. Furthermore, replantation was more likely to be cost-effective in younger patients (<48 years of age) and those with preinjury wages less than $42 000 per year. For certain older patients with anticipated shorter lifespans and higher costs of recovery, revision amputation may be a more cost-effective choice. Single-finger amputations had a lower probability of being cost-effective after replantation compared with multifinger amputations. However, the results were sensitive to the variability in postsurgical utility scores and wages. Our findings highlight the importance of careful patient selection based on finger amputation pattern, expected postsurgery utility, anticipated recovery time until return to work, and comorbidities to assess the potential cost-effectiveness after replantation.

In addition to age at time of injury, life expectancy, and preinjury wages, 2-way sensitivity analyses revealed that minimizing time off work, maximizing postinjury wages, and higher utility gain after replantation vs revision amputation were associated with cost-effectiveness. Because a large proportion of the total cost was attributable to postsurgery wages, the perioperative direct cost associated with complications did not substantially affect the overall cost-effectiveness of replantation. Motivated patients who want to return to work sooner and actively participate in hand therapy may have increased probability of more cost-effective replantation by optimizing time off work and increasing postsurgical salaries.

On the basis of the willingness-to-pay threshold of $100 000 per QALY, our study suggests that all traumatic finger amputation patterns, including single-finger injuries, may be cost-effective.^39,40^ Depending on the age at time of injury and life expectancy, presurgery and postsurgery salary, and time off work for recovery, the optimal intervention for traumatic finger amputation may vary. This finding highlights the importance of patient selection in traumatic amputation management before performing replantation or revision amputation.

### Strengths and Limitations

One of the strengths of the current study is that the economic analysis was based on a sample of real-world patients who underwent traumatic finger amputations. Another strength of our study is that patients were assessed with the SF-36 at least 1 year after their latest hand surgery, which should ensure incorporation of long-term utility after the acute perioperative changes of quality of life have resolved.

This study has limitations. An inherent limitation of the SF-36–derived utilities is that the SF-36 does not directly assess body image or psychological trauma that results from a traumatic finger amputation. However, traditional methods of eliciting utilities, such as the standard gamble and time trade-off for non–life-threatening disease processes, may yield inflated utility values.^41^ Future studies should use data from the National Hand Trauma Center Network^42^ to determine utilities that better reflect the psychological sequelae of traumatic finger amputations by using other methods, such as the discrete choice experiment.^43^ Despite differences in eliciting utilities, we found similar results as the prior study^10^ that replantation cost-effectiveness may be associated with injury patterns.

In addition, we assumed that long-term SF-36 values after replantation or revision amputation are stable 1 year after surgery. More nonwhite patients underwent replantation than white patients, although we found that the utility scores between the 2 racial groups were not significantly different. Ideally, utilities and model parameters should be based on randomized clinical trials, but to our knowledge, there are no randomized clinical trials comparing finger replantation with revision amputation because of ethical reasons. Consequently, the best evidence for this condition is a multicenter study, such as FRANCHISE. Because of the lack of randomization, there is a potential for confounding bias, but our model attempted to overcome such uncertainty through sensitivity analyses. Because of the relative rarity of certain traumatic finger amputation patterns, some injury pattern subgroups had small sample sizes. Our model excluded ambulance costs to the hospital and emergency department fees because we assumed that almost all patients, regardless of their eventual surgical management, initially had similar ambulance costs and were triaged in the emergency department. In addition, transportation costs to clinic visits were excluded because we assumed that the wages lost secondary to time off work would render transportation costs negligible. Also, emergency department fees were excluded in our analysis because patients in both cohorts likely were billed similar amounts for emergency services.

## Conclusions

Replantation of all finger amputation patterns, whether single-finger or multifinger injuries, may be cost-effective with proper patient selection. Because cost-effectiveness was associated with injury pattern and patient characteristics, if replantation is technically feasible, a thorough patient assessment and interview appear to be advisable before the surgeon chooses a treatment modality. Cost-effectiveness should not be the sole basis of clinical decision-making; however, cost-effectiveness information combined with patient-reported outcomes and clinical outcomes should be the foundation for a national traumatic finger amputation management guideline.

## Notes

Supplement. **eTable 1.** Descriptive Statistics of Patients by Injury Pattern **eTable 2.** One-Way Sensitivity Analyses **eFigure.** Cost-effectiveness Plane
