# Supplementary material for: Cost-effectiveness of Finger Replantation Compared With Revision Amputation
Source: JAMA Netw Open. 2019 Dec 2;2(12):e1916509. doi: 10.1001/jamanetworkopen.2019.16509 (PMC6902751; doi:10.1001/jamanetworkopen.2019.16509)
Supplement: Supplement. — eTable 1. Descriptive Statistics of Patients by Injury Pattern eTable 2. One-Way Sensitivity Analyses eFigure. Cost-effectiveness Plane [file jamanetwopen-2-e1916509-s001.pdf]

Supplementary Online Content

Yoon AP, Mahajani T, Hutton DW, Chung KC; Finger Replantation and Amputation Challenges in Assessing Impairment, Satisfaction, and Effectiveness (FRANCHISE) Group. Cost-effectiveness of finger replantation compared with revision amputation. *JAMA Netw Open.* 2019;2(12):e1916509. doi:10.1001/jamanetworkopen.2019.16509

eTable 1. Descriptive Statistics of Patients by Injury Pattern

eTable 2. One-Way Sensitivity Analyses

eFigure. Cost-effectiveness Plane

This supplementary material has been provided by the authors to give readers additional information about their work.

© 2019 Yoon AP et al. *JAMA Network Open*.

eTable 1. Descriptive Statistics of Patients by Injury Pattern

	Single Finger, not thumb (n=114)		P-value [a]	Thumb only (n=48)		P-value [a]	Multi-finger, not thumb (n=76)		P-value [a]	Multi-finger with thumb (n=19)		P-value [a]
	Rev amp	Replant		Rev amp	Replant		Rev amp	Replant		Rev amp	Replant	
	(n=47)	(n=67)		(n=12)	(n=36)		(n=17)	(n=59)		(n=3)	(n=16)	
**Age (years), mean (SD)**	52.1 (16.4)	45.6 (15.8)	0.04*	49.2 (19.6)	50.0 (13.9)	0.87	47.0 (15.9)	42.5 (14.0)	0.27	32.3 (16.0)	43.8 (15.9)	0.29
**Gender, No. (%)**												
**Male**	40 (85)	57 (85)	1.00	10 (83)	33 (92)	0.59	14 (82)	49 (83)	1.00	3 (100)	15 (94)	1
**Female**	7 (15)	10 (15)		2 (17)	3 (8)		3 (18)	10 (17)		0 (0)	1 (6)	
**Education, No. (%)**												
**Less than High school**	12 (26)	18 (27)	0.49	0 (0)	16 (44)	0.01*	8 (47)	17 (29)	0.76	3 (100)	5 (31)	0.75
**High school/GED**	12 (26)	27 (40)		4 (33)	8 (22)		5 (29)	20 (34)		0 (0)	3 (19)	
**Vocational/Technical School**	8 (17)	6 (9)		2 (17)	1 (3)		0 (0)	2 (3)		0 (0)	3 (19)	
**Some college**	4 (8)	3 (5)		1 (8)	3 (8)		1 (6)	6 (10)		0 (0)	1 (5)	
**College or more**	7 (15)	9 (13)		5 (42)	7 (19)		2 (12)	12 (20)		0 (0)	2 (13)	
**Unknown**	4 (8)	4 (6)		0 (0)	1 (3)		1 (6)	2 (3)		0 (0)	2 (13)	
**Race, No. (%)**												
**White**	10 (21)	0 (0)	<0.001*	7 (58)	8 (22)	0.03*	4 (24)	6 (10)	0.22	0 (0)	5 (31)	0.53
**Non-white**	37 (79)	67 (100)		5 (42)	28 (78)		13 (76)	53 (90)		3 (100)	11 (69)	
**Insurance, No. (%)**												
**Yes**	39 (83)	60 (90)	0.4	12 (100)	27 (75)	0.09	14 (82)	46 (78)	1.00	1 (33)	12 (75)	0.22
**No**	8 (17)	7 (10)		0 (0)	9 (25)		3 (18)	13 (22)		2 (67)	4 (25)	

[a] Differences in means for continuous variables was assessed using t-tests; categorical variables were assessed using chi-square tests or Fisher's exact test
[1] Rev amp: revision amputation
* Statistical significance (defined p <0.05)

© 2019 Yoon AP et al. *JAMA Network Open.*

eTable 2. One-Way Sensitivity Analyses

	Value		Single Digit, not Thumb		Thumb only		Multi-digit, Not Thumb		Multi-digit, including thumb	
**Parameter**	**Low**	**High**	**Low**	**High**	**Low**	**High**	**Low**	**High**	**Low**	**High**
**Age at time of injury**	18	79	$76,651	$188,727	$51,235	$120,245	$14,214	$35,366	$16,642	$39,881
**Complication Rates**										
**Revision**										
**Neuroma excision**	0	0.3	$100,656	$93,604	$67,412	$62,078	$18,657	$17,388	$21,878	$20,232
**Vascular re-exploration**	0	0	$99,157	$99,157	$66,278	$66,278	$18,388	$18,388	$21,528	$21,528
**Bone Procedure**	0	0	$99,157	$99,157	$66,278	$66,278	$18,388	$18,388	$21,528	$21,528
**Tendon Procedure**	0	0.15	$102,611	$95,703	$68,891	$63,665	$19,009	$17,766	$22,335	$20,722
**Minor revision procedure**	0	0.1	$99,630	$99,026	$66,636	$66,179	$18,473	$18,364	$21,639	$21,498
**Replantation**										
**Neuroma excision**	0	0.1	$98,442	$100,793	$65,738	$67,516	$18,259	$18,682	$21,361	$21,910
**Vascular re-exploration**	0	0.4	$96,813	$103,545	$64,505	$69,597	$17,966	$19,177	$20,981	$22,552
**Bone Procedure**	0	0.1	$96,422	$99,843	$64,210	$66,797	$17,896	$18,511	$20,890	$21,688
**Tendon Procedure**	0	0.3	$91,839	$105,657	$60,743	$71,195	$17,071	$19,557	$19,820	$23,046
**Minor revision procedure**	0	0.25	$98,077	$99,588	$65,461	$66,604	$18,193	$18,465	$21,276	$21,629
**Costs**										
**Revision**										
**Direct Costs**										
**Physician Fee**										
**Single Digit, not thumb**	597.55	808.45	$99,512	$98,802	$66,278	$66,278	$18,388	$18,388	$21,528	$21,528
**Thumb only**	597.55	808.45	$99,157	$99,157	$66,547	$66,010	$18,388	$18,388	$21,528	$21,528
**Multi-digit, not thumb**	1791.8	2424.2	$99,157	$99,157	$66,278	$66,278	$18,579	$18,196	$21,528	$21,528
**Multi-digit, including thumb**	1791.8	2424.2	$99,157	$99,157	$66,278	$66,278	$18,388	$18,388	$21,777	$21,280
**Anesthesia Fees**	221	299	$99,288	$99,026	$66,378	$66,179	$18,411	$18,364	$21,559	$21,498
**Hospital (facility?) Fees**	2389.35	3232.65	$100,578	$97,736	$67,353	$65,204	$18,643	$18,132	$21,860	$21,197
**Medication Cost**	522.75	707.25	$99,468	$98,846	$66,513	$66,043	$18,444	$18,332	$21,601	$21,456
**Physical Therapy Cost**										
**Single Digit, not thumb**	2109.7	2854.3	$100,412	$97,902	$66,278	$66,278	$18,388	$18,388	$21,528	$21,528
**Thumb only**	5018.4	6789.6	$99,157	$99,157	$68,536	$64,021	$18,388	$18,388	$21,528	$21,528
**Multi-digit, not thumb**	5854.8	7921.2	$99,157	$99,157	$66,278	$66,278	$19,014	$17,761	$21,528	$21,528
**Multi-digit, including thumb**	8364	11316	$99,157	$99,157	$66,278	$66,278	$18,388	$18,388	$22,689	$20,367
**Family Member Wages Lost**										
**Single Digit, not thumb**	1837.7	2486.3	$100,250	$98,064	$66,278	$66,278	$18,388	$18,388	$21,528	$21,528
**Thumb only**	1837.7	2486.3	$99,157	$99,157	$67,105	$65,452	$18,388	$18,388	$21,528	$21,528
**Multi-digit, not thumb**	2425.05	3280.95	$99,157	$99,157	$66,278	$66,278	$18,647	$18,128	$21,528	$21,528
**Multi-digit, including thumb**	2425.05	3280.95	$99,157	$99,157	$66,278	$66,278	$18,388	$18,388	$21,865	$21,192
**Replantation**										
**Direct Costs**										
**Physician Fee**										
**Single Digit, not thumb**	1941.4	2626.6	$98,003	$100,312	$66,278	$66,278	$18,388	$18,388	$21,528	$21,528

© 2019 Yoon AP et al. *JAMA Network Open*.

Thumb only	1941.4	2626.6	$99,157	$99,157	$65,405	$67,152	$18,388	$18,388	$21,528	$21,528
Multi-digit, not thumb	5825.05	7880.95	$99,157	$99,157	$66,278	$66,278	$17,764	$19,011	$21,528	$21,528
Multi-digit, including thumb	5825.05	7880.95	$99,157	$99,157	$66,278	$66,278	$18,388	$18,388	$20,720	$22,337
Anesthesia Fees										
Single Digit, not thumb	382.5	517.5	$99,157	$99,157	$66,278	$66,278	$18,388	$18,388	$21,528	$21,528
Thumb only	382.5	517.5	$99,157	$99,157	$66,278	$66,278	$18,388	$18,388	$21,528	$21,528
Multi-digit, not thumb	543.15	734.85	$99,157	$99,157	$66,278	$66,278	$18,388	$18,388	$21,528	$21,528
Multi-digit, including thumb	543.15	734.85	$99,157	$99,157	$66,278	$66,278	$18,388	$18,388	$21,528	$21,528
Hospital Fees	12526.45	16947.55	$91,708	$106,606	$60,644	$71,913	$17,047	$19,728	$19,790	$23,267
Medication Cost	522.75	707.25	$98,846	$99,468	$66,043	$66,513	$18,332	$18,444	$21,456	$21,601
Physical Therapy Cost										
Single Digit, not thumb	6119.15	8278.85	$95,518	$102,796	$66,278	$66,278	$18,388	$18,388	$21,528	$21,528
Thumb only	6119.15	8278.85	$99,157	$99,157	$63,526	$69,031	$18,388	$18,388	$21,528	$21,528
Multi-digit, not thumb	8364	11316	$99,157	$99,157	$66,278	$66,278	$17,493	$19,283	$21,528	$21,528
Multi-digit, including thumb	8364	11316	$99,157	$99,157	$66,278	$66,278	$18,388	$18,388	$20,367	$22,689
Family Member Wages Lost										
Single Digit, not thumb	2425.05	3280.95	$97,715	$100,599	$66,278	$66,278	$18,388	$18,388	$21,528	$21,528
Thumb only	2425.05	3280.95	$99,157	$99,157	$65,187	$67,369	$18,388	$18,388	$21,528	$21,528
Multi-digit, not thumb	2425.05	3280.95	$99,157	$99,157	$66,278	$66,278	$18,128	$18,647	$21,528	$21,528
Multi-digit, including thumb	2425.05	3280.95	$99,157	$99,157	$66,278	$66,278	$18,388	$18,388	$21,192	$21,865
Complication Costs										
Neuroma excision										
physician fee	589.05	796.95	$99,169	$99,145	$66,287	$66,269	$18,390	$18,386	$21,531	$21,526
anesthesia fee	141.1	190.9	$99,160	$99,154	$66,280	$66,276	$18,388	$18,387	$21,529	$21,528
hospital fee	4180.3	5655.7	$99,240	$99,074	$66,341	$66,216	$18,403	$18,373	$21,548	$21,509
physical tx/rehab	357.85	484.15	$99,164	$99,150	$66,284	$66,273	$18,389	$18,386	$21,530	$21,527
family member wages lost	661.3	894.7	$99,170	$99,144	$66,288	$66,268	$18,390	$18,385	$21,531	$21,525
Vascular re-exploration										
physician fee	1169.6	1582.4	$99,060	$99,254	$66,205	$66,352	$18,370	$18,405	$21,506	$21,551
anesthesia fee	382.5	517.5	$99,125	$99,189	$66,254	$66,302	$18,382	$18,393	$21,521	$21,536
hospital fee	2251.65	3046.35	$98,971	$99,344	$66,137	$66,419	$18,354	$18,421	$21,485	$21,572
physical tx/rehab	0	0	$99,157	$99,157	$66,278	$66,278	$18,388	$18,388	$21,528	$21,528
family member wages lost	441.15	596.85	$99,121	$99,194	$66,251	$66,306	$18,381	$18,394	$21,520	$21,537
Bone Procedure										
physician fee	917.15	1240.85	$99,113	$99,201	$66,245	$66,311	$18,380	$18,395	$21,518	$21,539
anesthesia fee	221	299	$99,147	$99,168	$66,270	$66,286	$18,386	$18,390	$21,526	$21,531
hospital fee	5064.3	6851.7	$98,916	$99,398	$66,096	$66,460	$18,344	$18,431	$21,472	$21,585
physical tx/rehab	0	0	$99,157	$99,157	$66,278	$66,278	$18,388	$18,388	$21,528	$21,528
family member wages lost	2425.05	3280.95	$99,042	$99,272	$66,191	$66,365	$18,367	$18,408	$21,501	$21,555
Tendon Procedure										
physician fee	673.2	910.8	$99,123	$99,191	$66,253	$66,304	$18,382	$18,394	$21,520	$21,536

© 2019 Yoon AP et al. *JAMA Network Open*.

anesthesia fee	221	299	$99,146	$99,168	$66,270	$66,287	$18,386	$18,390	$21,526	$21,531
hospital fee	5064.3	6851.7	$98,904	$99,410	$66,087	$66,469	$18,342	$18,433	$21,469	$21,587
physical tx/rehab	1984.75	2685.25	$99,058	$99,256	$66,203	$66,353	$18,370	$18,405	$21,505	$21,551
family member wages lost	3674.55	4971.45	$98,974	$99,340	$66,140	$66,417	$18,355	$18,421	$21,486	$21,571
Minor revision procedure										
physician fee	83.3	112.7	$99,152	$99,162	$66,275	$66,282	$18,387	$18,389	$21,527	$21,529
anesthesia fee	141.1	190.9	$99,149	$99,165	$66,272	$66,285	$18,386	$18,389	$21,526	$21,530
hospital fee	280.5	379.5	$99,140	$99,174	$66,266	$66,291	$18,385	$18,391	$21,524	$21,532
physical tx/rehab	357.85	484.15	$99,136	$99,178	$66,262	$66,294	$18,384	$18,391	$21,523	$21,533
family member wages lost	661.3	894.7	$99,118	$99,197	$66,248	$66,308	$18,381	$18,395	$21,519	$21,538
Wage Calculations										
Average annual mean wage before treatment	10000	250000	$79,609	$234,714	$51,492	$168,811	$14,870	$42,782	$16,965	$53,169
Time off for recovery and rehab (days)										
Revision	28	10000	$107,332	Dominant	$72,462	Dominant	$19,859	Dominant	$23,436	Dominant
Replantation	90	10000	$88,009	$3,770,455	$57,846	$2,843,182	$16,382	$679,051	$18,926	$878,464
Reduction in Wages following										
Revision	0	1	Dominant	$627,206	Dominant	$465,685	Dominant	$113,412	Dominant	$144,783
Replantation	0	1	$1,683,303	Dominant	$1,264,498	Dominant	$303,460	Dominant	$391,291	Dominant
Retirement age	57.8	78.2	$99,157	$99,157	$66,278	$66,278	$18,388	$18,388	$21,528	$21,528
Discount Rate	0.00001	0.05	$62,551	$127,787	$41,810	$85,415	$11,600	$23,697	$13,581	$27,744
Utilities										
Revision										
Single Digit, not thumb	0.807413	0.883055	$25,888	Dominated	$66,278	$66,278	$18,388	$18,388	$21,528	$21,528
Thumb only	0.774448	0.867052	$99,157	$99,157	$18,305	Dominated	$18,388	$18,388	$21,528	$21,528
Multi-digit, not thumb	0.66158	0.805361	$99,157	$99,157	$66,278	$66,278	$9,343	$576,617	$21,528	$21,528
Multi-digit, including thumb	0.601486	0.880514	$99,157	$99,157	$66,278	$66,278	$18,388	$18,388	$6,264	Dominated
Replantation										
Single Digit, not thumb	0.831025	0.886169	Dominated	$32,369	$66,278	$66,278	$18,388	$18,388	$21,528	$21,528
Thumb only	0.794591	0.882242	$99,157	$99,157	Dominated	$19,042	$18,388	$18,388	$21,528	$21,528
Multi-digit, not thumb	0.769093	0.846365	$99,157	$99,157	$66,278	$66,278	$38,331	$12,095	$21,528	$21,528
Multi-digit, including thumb	0.716107	0.880393	$99,157	$99,157	$66,278	$66,278	$18,388	$18,388	Dominated	$8,842

© 2019 Yoon AP et al. *JAMA Network Open*.

eFigure. Cost-effectiveness Plane

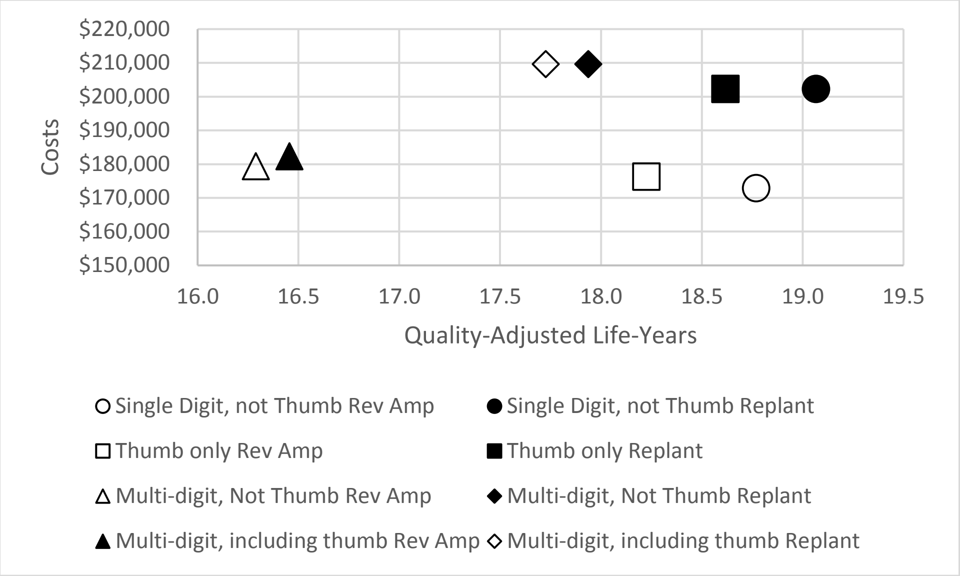

p

© 2019 Yoon AP et al. *JAMA Network Open*.
